# An Anatomical Cadaveric Demonstration of an Ultrasound-Guided Fascial Plane Injection Pathway in the Deep Gluteal Space

**DOI:** 10.3390/bioengineering13040412

**Published:** 2026-03-31

**Authors:** Sang-Hyun Kim, U-Young Lee, Yonghyun Yoon, Jihyo Hwang, Jonghyeok Lee, Seungbeom Kim, King Hei Stanley Lam, Teinny Suryadi, Anwar Suhaimi

**Affiliations:** 1 College of Korean Medicine, Woosuk University, 443, Samnye-ro, Samnye-eup, Wanju-gun 55338, Republic of Korea; amalang@naver.com; 2 Department of Anatomy, Catholic Institute for Applied Anatomy, College of Medicine, The Catholic University of Korea, Seoul 06649, Republic of Korea; cicloaum@catholic.ac.kr; 3 Department of Orthopaedic Surgery, Gangnam Sacred Heart Hospital, Hallym University College of Medicine, 1 Singil-ro, Yeongdeungpo-gu, Seoul 07441, Republic of Korea; 4 Incheon Terminal Orthopedic Surgery Clinic, Inha-ro 489beon-gil, Namdong-gu, Incheon 21574, Republic of Korea; 5 International Academy of Regenerative Medicine, Inha-ro 489beon-gil, Namdong-gu, Incheon 21574, Republic of Korea; stplayer@naver.com; 6 Board of Clinical Research, The International Association of Musculoskeletal Medicine, Kowloon, Hong Kong; drlamkh@gmail.com (K.H.S.L.); painfreedoc22@gmail.com (T.S.); anwar@ummc.edu.my (A.S.); 7 MSKUS, 1035 E. Vista Way #128, Vista, CA 92084, USA; 8 Bareun Neurosurgery Clinic, 39, Daenong-ro, Heungdeok-gu, Cheongju-si 28402, Republic of Korea; 9 Miso Pain Clinic, 1569, Bongyeong-ro, Yeongtong-gu, Suwon-si 16703, Republic of Korea; 10 Faculty of Medicine, The University of Hong Kong, Kowloon, Hong Kong; 11 Faculty of Medicine, The Chinese University of Hong Kong, New Territory, Hong Kong; 12 Department of Physical Medicine and Rehabilitation, Medistra Hospital, South Jakarta 12950, Indonesia; 13 Department of Physical Medicine and Rehabilitation, Synergy Clinic, West Jakarta 11470, Indonesia; 14 Department of Rehabilitation Medicine, Universiti Malaya, Kuala Lumpur 50603, Malaysia

**Keywords:** deep gluteal syndrome, fascial plane hydrodissection, ultrasound-guided intervention, sciatic nerve, enthesopathy, cadaveric feasibility

## Abstract

**Background:** Deep gluteal syndrome (DGS) has traditionally been attributed to sciatic nerve entrapment within the deep gluteal space. However, increasing evidence suggests that enthesopathy and soft tissue pathology of the short external rotators may also contribute to its pathogenesis. Conventional ultrasound-guided interventions primarily target the sciatic nerve through perineural hydrodissection (HD), which may not address enthesis-related pathology. However, the anatomical feasibility of delivering injectate along the deep gluteal fascial plane has not yet been investigated in cadaveric studies. **Methods:** This cadaveric anatomical demonstration evaluated whether an ultrasound-guided fascial plane injection within the deep gluteal space could simultaneously reach the enthesis of the short external rotators and the region of the sciatic nerve. Ultrasound scanning protocols were first demonstrated in a healthy volunteer to establish anatomical orientation for the injection pathway. Injection experiments were then performed in a fresh-frozen cadaver (83-year-old male) using a cranial-to-caudal in-plane approach. Ten milliliters of methylene blue dye was injected along the fascial plane overlying the short external rotator enthesis, followed by layer-by-layer cadaveric dissection to assess dye distribution. **Results:** Cadaveric dissection demonstrated that methylene blue injected along the deep gluteal fascial plane extended to the enthesis of the short external rotators and spread toward the surface of the sciatic nerve. Comparable distribution patterns were observed in both hips. These findings suggest that a single ultrasound-guided fascial plane injection trajectory may anatomically access both the enthesis region and the adjacent sciatic nerve within the deep gluteal space. **Conclusions:** Ultrasound-guided fascial plane HD in the deep gluteal space provides an anatomical pathway that can simultaneously access the enthesis of the short external rotators and the region of the sciatic nerve. This approach may represent a potential anatomical basis for a fascial plane-based intervention strategy in DGS. Further studies are required to evaluate in vivo behavior and clinical effectiveness.

## 1. Introduction

The term deep gluteal syndrome (DGS) was first introduced by McCrory and Bell to describe sciatic pain caused by compression of the sciatic nerve (SN) within the deep gluteal space [1]. In the literature, DGS is generally defined as compression or irritation of the SN in the deep gluteal space and is considered a non-discogenic cause of sciatic pain [2]. However, increasing anatomical and clinical evidence suggests that DGS is often multifactorial rather than solely a nerve entrapment disorder. Pathologies involving the short external rotators, proximal hamstring origin, and surrounding ligamentous structures may act as primary pain generators, with secondary neural irritation occurring as a consequence of enthesopathy, inflammation, or altered fascial tension [3,4].

The deep gluteal space is anatomically confined by the posterior acetabulum anteriorly, the gluteus maximus posteriorly, the sacrotuberous ligament medially, and the proximal hamstring origin inferiorly [5,6]. Within this compact region lie the piriformis, obturator internus–gemelli complex, quadratus femoris, and the SN in proximity [7,8,9]. Because of this spatial relationship, pathological changes at the tendon–bone interface or fascial planes may influence both musculoskeletal structures and adjacent neural elements [3].

Recent studies, including work from our research group, have suggested that DGS may frequently involve enthesopathy-related conditions affecting the short external rotators or adjacent ligamentous structures [10,11,12]. These findings imply that treatment strategies focusing exclusively on the SN may not fully address the underlying pathology in selected patients. Conventional ultrasound-guided SN hydrodissection (HD) primarily targets the perineural space to restore nerve mobility, but it may not directly influence the enthesis or interfascial pathology that may contribute to nerve irritation [13,14]. However, the anatomical feasibility of delivering injectate along this deep gluteal fascial plane has not yet been investigated in cadaveric studies.

Based on this concept, we hypothesized that HD performed within the deep gluteal fascial plane may reach both the enthesis of the short external rotators and the nearby SN environment. Therefore, this cadaveric anatomical demonstration aimed to evaluate whether a single ultrasound-guided fascial plane injection trajectory could anatomically reach both structures within the deep gluteal space.

## 2. Materials and Methods

### 2.1. Ethical Approval

This cadaveric study was reviewed by the Institutional Review Board of the Catholic University of Korea and was granted an exemption from full ethical review, as it involved the use of cadaveric specimens only and did not include human participants or identifiable personal data (IRB No. MIRB-면20260120-006).

### 2.2. Cadaver Preparation

The injection experiment was performed using one fresh-frozen cadaver (83-year-old male). The specimen was thawed at room temperature prior to the procedure. The cadaver was placed in a prone position, with the hip placed in internal rotation to facilitate exposure of the deep gluteal space and improve visualization of the short external rotators during ultrasound-guided injection.

Both hips were used for the injection procedure, followed by anatomical dissection to evaluate the distribution of methylene blue within the deep gluteal space.

It should be noted that cadaveric tissue properties, including reduced hydration and altered elasticity compared to living tissue, may influence injectate dispersion.

### 2.3. Ultrasonography

To demonstrate the ultrasonographic anatomy of the deep gluteal region, ultrasound images used to demonstrate the scanning protocol were obtained from a healthy volunteer after written informed consent was obtained.

Ultrasonographic evaluation was performed using an Alpinion XC90 Elite ultrasound system (ALPINION MEDICAL SYSTEMS Co., Ltd., Seoul, Republic of Korea) equipped with a linear transducer. Imaging parameters were standardized with a depth setting of 4 cm and a dynamic range of 60 dB.

Two scanning protocols were used to identify the deep gluteal structures.

First, the transducer was positioned at the level of the greater trochanter (GT) in an oblique sagittal plane and translated medially to visualize the short external rotators and adjacent structures.

Second, the probe was positioned over the greater sciatic notch and translated from cranial to caudal to sequentially visualize the piriformis muscle, sacrospinous ligament, obturator internus–gemelli complex, quadratus femoris, and proximal hamstring origin.

These scanning protocols were used to establish anatomical orientation for the ultrasound-guided injection approach.

### 2.4. Ultrasound-Guided Injection

Under real-time ultrasound guidance, a cranial-to-caudal in-plane approach was used to access the deep gluteal space, consistent with established principles of ultrasound-guided musculoskeletal interventions [15]. The transducer was positioned just medial to the GT at a level where the short external rotators could be simultaneously visualized in the short axis plane. The needle entry point was selected at the region where the osseous acoustic shadow from the GT disappeared, allowing unobstructed access to the deep gluteal fascial plane.

A 23-gauge, 6 cm needle was advanced from cranial to caudal in a posterior-to-anterior direction under continuous visualization of the needle shaft and tip. At approximately 4 cm depth, the needle tip was positioned over the enthesis of the short external rotators. Methylene blue (10 mL) was then slowly injected as a visual tracer while monitoring dispersion within the deep gluteal fascial plane.

Methylene blue was selected to allow clear identification of injectate distribution during subsequent anatomical dissection. Methylene blue is widely used as a visual tracer in cadaveric injection studies to facilitate direct visualization of injectate distribution during anatomical dissection [16]. However, due to its lower viscosity and higher diffusion properties compared to clinical injectates, its distribution pattern should be interpreted with caution. The trajectory of the needle and distribution of the injectate were continuously observed to assess coverage of the enthesis and the interfascial plane. The SN was visualized as an adjacent anatomical landmark during the procedure but was not directly targeted. All ultrasound procedures were performed by an orthopedic surgeon with more than 10 years of experience in musculoskeletal ultrasonography.

### 2.5. Anatomical Dissection

Anatomical dissection was performed by an anatomist with more than 10 years of experience in cadaveric dissection using a meticulous layer-by-layer posterior approach. Following injection, the skin and subcutaneous tissues were removed, followed by detachment of the gluteus maximus and gluteus medius to expose the deep gluteal structures. The short external rotators were then identified.

The distribution of methylene blue dye was documented with particular attention to whether the injectate reached the enthesis of the short external rotators and whether it extended to the surface of the SN.

## 3. Results

### 3.1. Ultrasonographic Identification of the Deep Gluteal Structures

**Figure 1:**
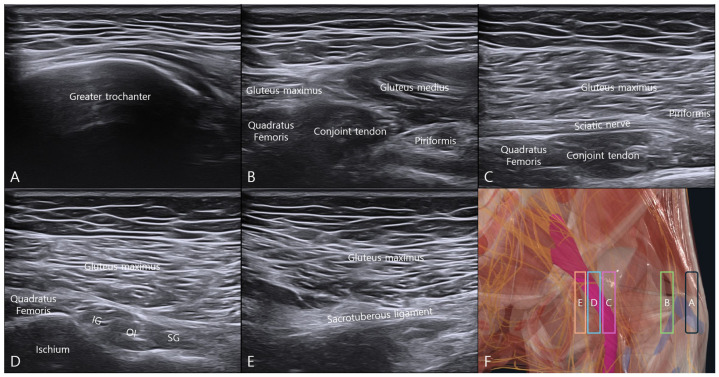
Ultrasonographic evaluation of the deep gluteal space using medial probe translation from the greater trochanter. The transducer was initially positioned in an oblique sagittal plane at the level of the greater trochanter (GT) and translated medially while maintaining the same imaging plane to sequentially visualize the deep gluteal structures. (**A**) Ultrasound image obtained at the level of the GT. (**B**) Medial translation of the probe from the GT while maintaining the oblique sagittal plane. (**C**) Visualization of the sciatic nerve in its longitudinal course with the short external rotators identified beneath it. (**D**) Visualization of the origin of the short external rotators over the ischium. (**E**) Further medial translation demonstrating the sacrotuberous ligament. (**F**) Schematic illustration indicating the probe positions corresponding to each ultrasound image. Abbreviations: SG, superior gemellus; OI, obturator internus; IG, inferior gemellus.

Using the GT as the initial landmark, medial probe translation enabled sequential visualization of the deep gluteal structures (Figure 1). The transducer was positioned in an oblique sagittal plane at the level of the GT, and medial translation progressively revealed the short external rotators and adjacent structures. With further medial translation, the SN became visible superficial to the short external rotators. At the level of the ischium, the origins of the short external rotators were identified, and additional medial translation revealed the sacrotuberous ligament. These findings established the anatomical orientation of the deep gluteal region relevant to the injection approach.

### 3.2. Cranial-to-Caudal Ultrasonographic Mapping of the Deep Gluteal Space

**Figure 2:**
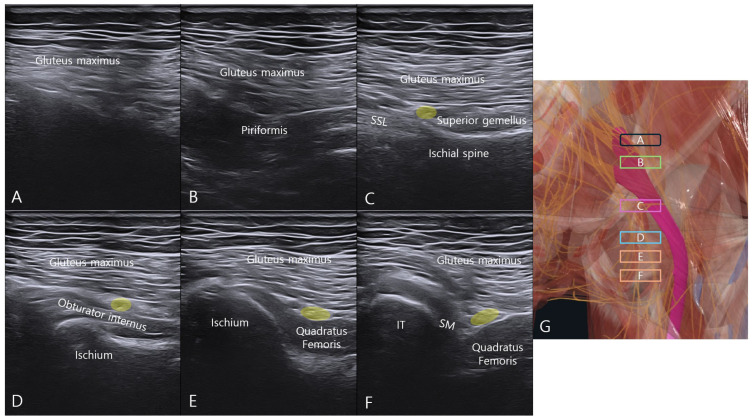
Ultrasonographic mapping of the deep gluteal space using cranial-to-caudal probe translation from the greater sciatic notch. The transducer was positioned over the greater sciatic notch and translated cranially to caudally to sequentially visualize the deep gluteal structures. (**A**) Cranial to the greater sciatic notch, where ultrasound penetration is limited by the overlying bony structure. (**B**) Greater sciatic notch level showing the piriformis deep to the gluteus maximus. (**C**) Ischial spine level demonstrating the sacrospinous ligament medially and superior gemellus laterally. (**D**) Obturator internus level with the sciatic nerve (yellow circle) between the gluteus maximus and obturator internus. (**E**) Quadratus femoris level with the sciatic nerve (yellow circle) between the gluteus maximus and quadratus femoris. (**F**) Level of the ischial tuberosity showing the proximal hamstring tendon (semimembranosus) with the sciatic nerve between the semimembranosus and quadratus femoris. (**G**) Schematic illustration of probe positions corresponding to each ultrasound image. Abbreviations: SSL, sacrospinous ligament; IT, ischial tuberosity; SM, semimembranosus; OI, obturator internus; QF, quadratus femoris.

Sequential cranial-to-caudal scanning from the greater sciatic notch demonstrated the layered anatomy of the deep gluteal space (Figure 2). Cranial to the greater sciatic notch, ultrasound penetration was limited by the overlying bony structure. At the greater sciatic notch level, the piriformis muscle was visualized deep to the gluteus maximus.

At the ischial spine level, the sacrospinous ligament was identified medially with the superior gemellus attached laterally. With further caudal translation, the SN was visualized between the gluteus maximus and the obturator internus and remained identifiable between the gluteus maximus and quadratus femoris at the quadratus femoris level [17].

Further caudal scanning revealed the appearance of the proximal hamstring tendon (semimembranosus) as the ischial tuberosity became visible, with the SN located between the semimembranosus and quadratus femoris. This cranial-to-caudal scanning protocol allowed consistent identification of the deep gluteal structures relevant to the injection pathway.

### 3.3. Ultrasound-Guided Injection Technique

**Figure 3:**
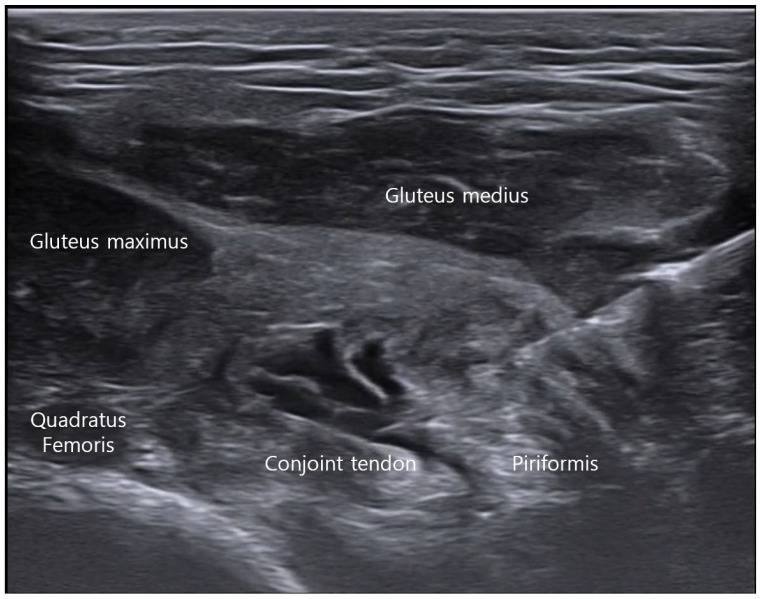
Ultrasound-guided injection into the deep gluteal fascial plane using a cranial-to-caudal in-plane approach. With the cadaver placed in a prone position, the needle was advanced from cranial to caudal using an in-plane technique under real-time ultrasound guidance. The injection was directed into the fascial plane between the short external rotators and the overlying gluteus medius and gluteus maximus muscles.

With the cadaver placed in a prone position, a cranial-to-caudal in-plane approach was used to access the deep gluteal fascial plane (Figure 3). Under real-time ultrasound guidance, the needle was advanced between the short external rotators and the overlying gluteal muscles. This trajectory enabled delivery of the injectate along the fascial plane overlying the enthesis of the short external rotators.

### 3.4. Cadaveric Assessment of Injectate Distribution

**Figure 4:**
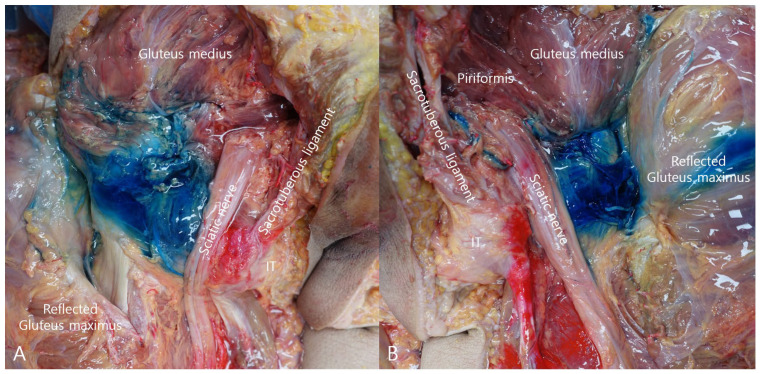
Cadaveric dissection demonstrating methylene blue dispersion within the deep gluteal space. In the prone cadaveric dissection, methylene blue dispersion was observed extending proximally toward the gluteal crease and medially toward the sciatic nerve. (**A**) Left hip showing methylene blue distribution within the deep gluteal space with staining extending to the surface of the sciatic nerve. (**B**) Right hip demonstrating methylene blue spread within the deep gluteal space and along the sciatic nerve.

Layer-by-layer cadaveric dissection demonstrated the distribution of methylene blue within the deep gluteal space (Figure 4). In both hips, methylene blue staining extended to the enthesis of the short external rotators and along the surface of the SN. These findings suggest that the injectate extended toward both the enthesis region and the perineural area within the deep gluteal space.

## 4. Discussion

The principal finding of this cadaveric anatomical demonstration is that a single ultrasound-guided injection performed within the deep gluteal fascial plane can anatomically access both the enthesis of the short external rotators and the region of the sciatic nerve within the deep gluteal space. Cadaveric dissection demonstrated that methylene blue injected along the fascial interface between the short external rotators and the overlying gluteal muscles extended toward the enthesis and spread to the surface of the sciatic nerve. These findings suggest that injectate delivered through this pathway may reach both the tendon–bone interface and the adjacent neural structures. This study provides an initial anatomical demonstration of a fascial-plane-based injection pathway in the deep gluteal space.

DGS has traditionally been considered a condition primarily caused by SN entrapment within the deep gluteal space [18]. Accordingly, many treatment strategies have focused on direct neural decompression, including ultrasound-guided perineural HD of the SN, targeted injections around the piriformis muscle, and even endoscopic surgical decompression of the SN within the deep gluteal space [19,20,21,22]. However, emerging anatomical and clinical evidence suggests that DGS frequently involves enthesopathy or soft tissue pathology affecting the short external rotators, ligaments, or proximal hamstring origin [10,11,12]. In such cases, SN irritation may represent a secondary phenomenon resulting from local inflammation, mechanical compression, or altered fascial tension. Therefore, treatment strategies directed solely at the SN may not fully address the underlying pathological process.

The present findings support the concept that HD performed along the deep gluteal fascial plane may provide a potential anatomical basis for a broader therapeutic strategy. By delivering injectate along the fascial interface overlying the enthesis of the short external rotators, this approach may potentially facilitate mechanical separation of fascial planes and influence the local tissue environment at the tendon–bone interface [13,14,23]. Because the injectate spreads toward the region of the SN, this technique may also provide indirect neural decompression without requiring direct perineural targeting.

This anatomical observation supports the feasibility of a fascial plane-based approach for ultrasound-guided intervention in DGS. Rather than targeting the SN alone, this approach allows simultaneous access to both the enthesis of the short external rotators and the nearby neural structures. Such a strategy may be particularly relevant in cases where enthesopathy and secondary neural irritation coexist, which has been increasingly suggested in recent studies of DGS pathophysiology [10,11,12].

From a therapeutic perspective, this injection pathway may also allow the application of regenerative injection therapy (RIT). RIT techniques—including prolotherapy, platelet-rich plasma, or other biologic injectates—aim to promote tissue repair and modulate chronic inflammatory processes at entheses or degenerative soft tissues [24,25]. Delivery of such injectates along the deep gluteal fascial plane may therefore combine mechanical HD with regenerative modulation of the enthesis while simultaneously affecting the surrounding neural environment.

The current study represents an initial cadaveric anatomical demonstration of this injection pathway. However, injectate dispersion may vary depending on injection volume and tissue characteristics. Future cadaveric studies involving larger specimen numbers are planned to investigate injectate distribution patterns further. In particular, comparative experiments using different injection volumes (e.g., 10 mL and 30 mL) will be conducted to evaluate how injectate spreads within the deep gluteal space and to determine the extent to which it reaches the SN and surrounding structures.

Several limitations should be acknowledged. First, this study was conducted using a single cadaver specimen, which limits generalizability. Nevertheless, cadaveric studies remain an important methodological approach for evaluating anatomical feasibility and injection pathways in musculoskeletal research, as highlighted in methodological quality assessments such as the QUACS scale [26]. Second, cadaveric tissue characteristics differ from those of living tissue, particularly with respect to tissue compliance and fluid dispersion. In particular, hydrodissection is a dynamic process that depends on tissue compliance and fluid behavior, which cannot be fully replicated under cadaveric conditions. Additionally, methylene blue dye was used as a visual tracer and may not accurately reflect the distribution characteristics of clinical injectates due to its lower viscosity and higher diffusion properties. Nevertheless, direct anatomical confirmation through layer-by-layer dissection provides valuable insight into the potential injection pathways within the deep gluteal space. Furthermore, the prone positioning used in this cadaveric model may not fully reflect physiological conditions, which could influence injectate distribution patterns.

Despite these limitations, the present study provides an anatomical basis for a fascial plane-based HD technique in the management of deep gluteal syndrome. Further studies involving larger cadaveric series and clinical outcome investigations will be necessary to determine the optimal injection volume, distribution patterns, and therapeutic effectiveness of this approach.

## 5. Conclusions

This cadaveric anatomical demonstration demonstrated that an ultrasound-guided injection performed within the deep gluteal fascial plane can reach both the enthesis of the short external rotators and the region of the SN through a single injection trajectory. Cadaveric dissection confirmed that injectate delivered along this fascial interface extended to the enthesis and spread toward the surface of the SN within the deep gluteal space.

These findings suggest that a fascial plan-based HD approach may provide a potential ultrasound-guided intervention strategy for deep gluteal syndrome. By accessing both the enthesis and the adjacent neural structures through a single injection pathway, this technique may have potential implications for combining mechanical HD with regenerative injection strategies.

Further cadaveric studies involving larger specimen numbers and varying injection volumes will be necessary to better understand injectate distribution patterns within the deep gluteal space and to determine the optimal parameters for clinical application.

## Data Availability

The data supporting the findings of this study are available within the article. Additional materials may be available from the corresponding author upon reasonable request.
